# Outcomes of Haploidentical Stem Cell Transplant Recipients With HHV-6B Reactivation

**DOI:** 10.1093/ofid/ofae564

**Published:** 2024-09-26

**Authors:** Guy Handley, Amanda Yepes, Eva Eliassen, Gabriel Dominguez, Yanina Pasikhova, Olga Klinkova, Aliyah Baluch, Anthony J Febres-Aldana, Melissa Alsina, Hany Elmariah, Farhad Khimani, Doris K Hansen, Ciara L Freeman, Michael D Jain, Frederick Locke, Aleksandr Lazaryan, Hein D Liu, Asmita Mishra, Abu-Sayeef Mirza, Taiga Nishihori, Leonel Ochoa, Lia Perez, Joseph Pidala, Omar Castaneda Puglianini, Michael Nieder, Fabiana Perna, Jongphil Kim, Nelli Bejanyan, Rawan Faramand

**Affiliations:** Division of Infectious Disease and International Medicine, Department of Internal Medicine, Morsani College of Medicine, University of South Florida, Tampa, Florida, USA; H. Lee Moffitt Cancer Center and Research Institute, Tampa, Florida, USA; Department of Pharmacy, H. Lee Moffitt Cancer Center and Research Institute, Tampa, Florida, USA; Morsani College of Medicine, University of South Florida, Tampa, Florida, USA; Morsani College of Medicine, University of South Florida, Tampa, Florida, USA; Department of Pharmacy, H. Lee Moffitt Cancer Center and Research Institute, Tampa, Florida, USA; H. Lee Moffitt Cancer Center and Research Institute, Tampa, Florida, USA; H. Lee Moffitt Cancer Center and Research Institute, Tampa, Florida, USA; H. Lee Moffitt Cancer Center and Research Institute, Tampa, Florida, USA; Department of Blood and Marrow Transplant and Cellular Immunotherapy, H. Lee Moffitt Cancer Center and Research Institute, Tampa, Florida, USA; Department of Blood and Marrow Transplant and Cellular Immunotherapy, H. Lee Moffitt Cancer Center and Research Institute, Tampa, Florida, USA; Department of Blood and Marrow Transplant and Cellular Immunotherapy, H. Lee Moffitt Cancer Center and Research Institute, Tampa, Florida, USA; Department of Blood and Marrow Transplant and Cellular Immunotherapy, H. Lee Moffitt Cancer Center and Research Institute, Tampa, Florida, USA; Department of Blood and Marrow Transplant and Cellular Immunotherapy, H. Lee Moffitt Cancer Center and Research Institute, Tampa, Florida, USA; Department of Blood and Marrow Transplant and Cellular Immunotherapy, H. Lee Moffitt Cancer Center and Research Institute, Tampa, Florida, USA; Department of Blood and Marrow Transplant and Cellular Immunotherapy, H. Lee Moffitt Cancer Center and Research Institute, Tampa, Florida, USA; Department of Blood and Marrow Transplant and Cellular Immunotherapy, H. Lee Moffitt Cancer Center and Research Institute, Tampa, Florida, USA; Department of Blood and Marrow Transplant and Cellular Immunotherapy, H. Lee Moffitt Cancer Center and Research Institute, Tampa, Florida, USA; Department of Blood and Marrow Transplant and Cellular Immunotherapy, H. Lee Moffitt Cancer Center and Research Institute, Tampa, Florida, USA; Department of Blood and Marrow Transplant and Cellular Immunotherapy, H. Lee Moffitt Cancer Center and Research Institute, Tampa, Florida, USA; Department of Blood and Marrow Transplant and Cellular Immunotherapy, H. Lee Moffitt Cancer Center and Research Institute, Tampa, Florida, USA; Department of Blood and Marrow Transplant and Cellular Immunotherapy, H. Lee Moffitt Cancer Center and Research Institute, Tampa, Florida, USA; Department of Blood and Marrow Transplant and Cellular Immunotherapy, H. Lee Moffitt Cancer Center and Research Institute, Tampa, Florida, USA; Department of Blood and Marrow Transplant and Cellular Immunotherapy, H. Lee Moffitt Cancer Center and Research Institute, Tampa, Florida, USA; Department of Blood and Marrow Transplant and Cellular Immunotherapy, H. Lee Moffitt Cancer Center and Research Institute, Tampa, Florida, USA; Department of Blood and Marrow Transplant and Cellular Immunotherapy, H. Lee Moffitt Cancer Center and Research Institute, Tampa, Florida, USA; Department of Blood and Marrow Transplant and Cellular Immunotherapy, H. Lee Moffitt Cancer Center and Research Institute, Tampa, Florida, USA; Department of Biostatistics, H. Lee Moffitt Cancer Center and Research Institute, Tampa, Florida, USA; Department of Blood and Marrow Transplant and Cellular Immunotherapy, H. Lee Moffitt Cancer Center and Research Institute, Tampa, Florida, USA; Department of Blood and Marrow Transplant and Cellular Immunotherapy, H. Lee Moffitt Cancer Center and Research Institute, Tampa, Florida, USA

**Keywords:** allogeneic hematopoietic stem cell transplant, haploidentical, HHV6, post-transplant cyclophosphamide, viral reactivations

## Abstract

**Background:**

Human herpesvirus 6B (HHV-6B) frequently reactivates following allogeneic stem cell transplant (alloHCT). Consensus guidelines note that haploidentical alloHCT may represent a high-risk population for which there is little evidence; this warrants further investigation.

**Methods:**

In this single-center retrospective study, we evaluated 188 consecutive adult patients receiving haploidentical alloHCT between 11/2014 and 11/2020 and compared outcomes between patients with HHV-6B reactivation receiving targeted antiviral therapy and those who were clinically observed.

**Results:**

Of the 58 included patients, 21 (36.2%) received antiviral therapy for HHV-6B reactivation with foscarnet (n = 19) or ganciclovir (n = 2). There were no differences in patient or disease characteristics between treated and observed patients. Treated patients were more likely to have high-level DNAemia (85.7% vs 40.5%; *P* < .001) and had higher peak viral quantitative measurements (median log_10_, 4.65 vs 3.84; *P* < .001). The median time to clearance from plasma (interquartile range) was 13 (7.25–20.00) days for all patients and was not significantly different between groups. There were no differences in episodes of encephalitis, grade III/IV acute graft-vs-host disease (GVHD), or time to neutrophil or platelet engraftment among treated vs observed patients. Day 100 nonrelapse mortality was not significantly different in the multivariate analysis; however, the presence of central nervous system symptoms was strongly associated with worse survival (hazard ratio, 4.11; 95% CI, 1.27–13.34; *P* = .018).

**Conclusions:**

We did not observe a difference in clinical outcomes between the treated and observed groups of patients with HHV-6B reactivation following haploidentical alloHCT. With the rising use of haploidentical transplant and post-transplant cyclophosphamide GVHD prevention platforms, prospective studies are needed to further characterize the risk and outcomes associated with HHV-6B reactivation and therapy.

Human herpesvirus 6 (HHV-6) reactivation regularly occurs following allogeneic stem cell transplant (alloHCT). The overall incidence of HHV-6 DNAemia is 30%–80% in alloHCT patients and typically occurs early, within the first 6 weeks after transplant [1–3]. Reactivation has been associated with increased all-cause mortality, delayed platelet engraftment, and grade III/IV acute graft-vs-host disease (GVHD) [1–4]. Two distinct viruses exist, HHV-6A and HHV-6B; however, after primary infection and telomeric integration, HHV-6A is typically transcriptionally silent, undergoing heavy histone modifications [5, 6]. The vast majority of HHV-6 reactivation and end-organ disease derives from HHV-6B. The typical natural history of HHV-6B reactivation following alloHCT is spontaneous resolution without sequelae [1, 7, 8].

HHV-6B reactivation can be associated with end-organ disease but requires exclusion of alternative diagnoses. Encephalitis carries the most evidence for a causal link to HHV-6B and may present with a well-defined post-transplant acute limbic encephalitis (PALE) with high mortality and devastating neurologic sequelae [9–13]. Other associations have been described, such as myelosuppression, pneumonitis, acute GVHD, fever with rash, hepatitis, or graft failure, with varying degrees of in vitro or in vivo evidence of correlation with HHV-6B [9]. A direct causal link in vivo may be difficult to establish and require exclusion of alternative diagnoses. Several studies have evaluated the use of preemptive or prophylactic therapy to prevent end-organ disease or HHV-6B reactivation and, unfortunately, have not shown clinical efficacy to prevent encephalitis with potential adverse effects from antiviral agents [14–20].

Certain types of transplants, such as umbilical cord blood transplantation, where reactivation rates may be >90%, carry increased risks of HHV-6B encephalitis; however, studies of prospectively monitored patients have not shown evidence that antiviral therapy mitigates this risk [3, 8]. As such, published guidelines from the European Conference on Infections in Leukemia (ECIL) and the Japan Society for Hematopoietic Cell Transplantation recommend against prospective monitoring and prophylactic or preemptive therapy of HHV-6B following routine alloHCT due to limited evidence of efficacy to prevent end-organ disease [9, 21]. Both guidelines note haploidentical transplantation as a potential high-risk population for HHV-6B encephalitis [9, 21]. There are limited data regarding the clinical course and nature of HHV-6B reactivation following haploidentical alloHCT. This retrospective analysis sought to characterize this risk, as well as evaluate the outcomes of patients who received antiviral therapy for HHV-6B reactivation.

## METHODS

### Study Design

This is a retrospective, single-center analysis of patients undergoing haploidentical alloHCT at Moffitt Cancer Center, a National Cancer Institute–designated Comprehensive Cancer Center. The institutional review board reviewed and approved the protocol. Clinical investigation was conducted according to the Declaration of Helsinki principles. All patients undergoing haploidentical alloHCT from 11/2014 to 11/2020 were identified. Patients with detection of HHV-6 DNA by plasma real-time polymerase chain reaction (PCR) were included in the study. Demographic, laboratory, radiographic, microbiologic, and clinical data including use of antiviral agents with activity against HHV-6B were extracted from the electronic medical record for up to 1 year after transplantation. Baseline characteristics for haploidentical alloHCT patients with HHV-6B reactivation were described and compared between treated patients and those observed clinically. The primary outcome was day 100 nonrelapse mortality (NRM) between patients treated for HHV-6B reactivation and those who were clinically observed. Secondary outcomes included evaluation of peak levels of DNAemia, duration of DNAemia, development of encephalitis, incidence of acute GVHD, and time to neutrophil and platelet engraftment as well as toxicities from antiviral therapy. Individual assessments for HHV-6 DNA in plasma were considered symptomatic if obtained for central nervous system (CNS) symptoms or dysfunction, fever, unexplained cytopenias, or rash. Assessments were considered serial monitoring if obtained at the treating physician's discretion in the absence of reported symptoms. This was typically weekly and occurred immediately following transplant in 15 of the 27 patients, while initiated later for other patients. CNS symptoms or dysfunction was defined as new altered mental status or a focal neurologic deficit. High-level DNAemia was defined as ≥10 000 copies/mL HHV-6B DNA in plasma based on historical literature. HHV-6B encephalitis was defined as new altered mental status with detection of HHV-6 DNA in the cerebrospinal fluid (CSF). The Sequential Organ Failure Assessment (SOFA) was utilized to determine clinical acuity and degree of organ dysfunction at the time of peak DNAemia to compare groups [22]. Acute kidney injury (AKI) was defined using a guideline published from the Kidney Disease Improving Global Outcomes Guidelines (KDIGO) clinical practice guidelines [23]. For 2 patients receiving supplemental oxygen without an arterial blood gas, the PaO2 was determined using a model to calculate a SOFA score [24]. GVHD was defined by consensus definitions [25].

### Institutional Protocol

Lumbar puncture (LP) was recommended for patients with a viral load of >10 000 copies/mL or in symptomatic patients to evaluate for HHV-6 DNA in the CSF at our institution. In patients with unexplained fever, rash, cytopenias, or confusion, treatment was recommended in the presence of HHV-6 DNA at >10 000 copies/mL. These recommendations were removed when the protocol was updated in December 2022 and left to physician discretion. Prospective monitoring of HHV-6B viral load by PCR and initiation of antiviral treatment were at the discretion of treating physicians.

### Assays Used

HHV-6 DNA was evaluated using quantitative PCR testing of plasma samples by commercially available testing that differentiated between HHV-6A and HHV-6B (ARUP Laboratories, Salt Lake City, UT, USA). CSF samples were evaluated by this PCR test and/or a qualitative nested multiplex PCR panel that did not differentiate between HHV-6A and HHV-6B (BioFire FilmArray Meningitis/Encephalitis Panel, BioFire Diagnostics, Salt Lake City, UT, USA).

### Statistical Analysis

Collected data are presented as medians and interquartile ranges (IQRs) for continuous variables and medians and percentages for ordinal variables. The Mann-Whitney *U* test was used for comparison of continuous variables, and bivariate associations were evaluated utilizing the Pearson chi-square test, or the Fisher exact test in the instance of not enough occurrences to utilize the chi-square test. A *P* value of <.05 was considered statistically significant. A univariate Cox proportional hazards model and a multivariate Cox proportional hazards model by backward elimination at a *P* value of <.1 were created to evaluate the relative risk. Hazard ratios (HRs) and 95% CIs are presented. Statistical analyses were conducted using SPSS Statistics, version 29.0.1.0 (SPSS, Inc., Chicago, IL, USA), and SAS, version 9.3 (SAS Institute, Cary, NC, USA).

## RESULTS

### Patient and Disease Characteristics

A total of 188 adults underwent haploidentical alloHCT during the study period. Of those, 121 patients (64.4%) had at least 1 HHV-6 measurement during the first 100 days after transplant. HHV-6 DNA was detected in 58 (47.9%) of these 121 patients who were included in the study analysis. All 58 cases identified had HHV-6B detected by plasma PCR testing. Antiviral therapy for HHV-6B reactivation was initiated in 21 (36.2%) patients. No significant differences in age, sex, performance status, race, malignancy diagnosis, graft source, conditioning regimen, GVHD prevention regimen, cytomegalovirus (CMV) serostatus, or SOFA scores at peak viral loads were noted (Table 1). The indication for obtaining HHV-6B DNA PCR levels, serial monitoring, or evaluation due to clinical symptoms did not differ between groups. Additional variables at onset of HHV-6B DNAemia are presented in Supplementary Table 1. At the onset of DNAemia, treated patients were more likely to have an absolute neutrophil count <500/mm^3^ (42.9% vs 5.4%; *P* < .001) and to not have achieved platelet engraftment (85.7% vs 59.5%; *P* = .038). No difference was detected in the rates of grade II–IV or grade III/IV acute GVHD between the groups at the onset of DNAemia.

**Table 1.** Characteristics of Treated vs Observed Patients With HHV-6B Reactivation

Baseline Characteristics	Observed (n = 37)	Treated (n = 21)	*P* Value
Median age (IQR), y	58 (46.0–66.5)	63 (26.0–66.5)	.948
Female, No. (%)	11 (29.7)	11 (52.4)	.088
Performance status, KPS ≥80, No. (%)	35 (94.6)	19 (90.5)	.615
SOFA score at peak viral load (IQR)	3 (2–4)	4 (3–5)	.128
Race, No. (%)			.776
White	26 (70.2)	14 (66.7)	
Not White	11 (29.8)	7 (33.3)	
Diagnosis, No. (%)			.211
AML	18 (48.6)	5 (23.8)	
ALL	2 (5.4)	4 (19)	
MDS	8 (21.6)	5 (23.8)	
Other	9 (24.4)^a^	7 (33.4)^b^	
Graft source, No. (%)			1.000
Peripheral blood	32 (86.4)	18 (85.7)	
Bone marrow	5 (13.6)	3 (14.3)	
Conditioning, No. (%)			.686
Myeloablative	13 (35.1)	9 (42.8)	
Reduced intensity	20 (54.0)	11 (52.4)	
Nonmyeloablative	4 (10.9)	1 (4.8)	
ATG use, No. (%)	5 (13.6)	2 (9.5)	1.000
GVHD regimen, No. (%)			.776
PTCy/tacrolimus/MMF	11 (29.7)	7 (33.3)	
PTCy/sirolimus/MMF	26 (70.3)	14 (66.7)	
CMV status, No. (%)			.775
D+/R+	17 (45.9)	11 (52.4)	
D+/R–	3 (8.1)	3 (14.3)	
D−/R+	10 (27.0)	4 (19.0)	
D−/R−	7 (19.0)	3 (14.3)	
HHV-6 testing, No. (%)			.503
Serial monitoring	16 (43.2)	11 (52.4)	
Symptomatic^c^	21 (56.8)	10 (47.6)	

Abbreviations: ALL, acute lymphoblastic leukemia; AML, acute myelogenous leukemia; ATG, antithymocyte globulin; CML, chronic myeloid leukemia; CMV, cytomegalovirus; GVHD, graft-vs-host disease; KPS, Karnofsky Performance Score; MDS, myelodysplastic syndrome; MMF, mycophenolate mofetil; MPS, myeloproliferative syndrome; PLL, prolymphocytic leukemia; PTCy, post-transplant cyclophosphamide; SOFA, Sequential Organ Failure Assessment.

^a^CML (n = 1), MPS (n = 1), sickle cell anemia (n = 2), Hodgkin's lymphoma (n = 2), aplastic anemia (n = 2), PLL (n = 1).

^b^CML (n = 1), MPS (n = 3), adrenoleukodystrophy (n = 1), sickle cell (n = 2).

^c^Neurologic dysfunction, fever, cytopenias, rash.

### HHV-6B Treatment

Of 21 treated patients, 19 (90.5%) received intravenous foscarnet, while 2 (9.5%) received intravenous ganciclovir. The median overall duration of treatment (IQR) was 18 (12–21) days. For the 19 patients receiving foscarnet therapy, 8 (42.1%) developed an AKI by KDIGO definitions after therapy was initiated. Antivirals were attributed as the causative etiology of AKI by treating physicians in 6 treated patients and were stopped entirely in 2 patients, while 1 was changed to intravenous ganciclovir. Of those, only 2 (25%) had recovery to baseline renal function.

### Characteristics of HHV-6B Reactivation and Patient Outcomes

Characteristics of HHV-6B reactivation and outcomes of treated vs observed patients are summarized in Table 2. Treated patients were more likely to have a high level of HHV-6B reactivation (85.7% vs 40.5%; *P* < .001) and a higher peak of viral quantitative measurements (median log_10_, 4.65 vs 3.84; *P* < .001). Overall, the median onset of DNAemia (IQR) was 25 (20.00–31.20) days but occurred earlier in treated patients (median, 23 vs 26 days; *P* = .022). Clearance of HHV-6B DNA in plasma during the initial episode of HHV-6B reactivation was documented in 52 (90.0%) patients, with a median time to clearance (IQR) of 13 (7–20) days, and was not significantly different between groups. Comparing treated and observed patients, the cumulative incidence of grade III/IV acute GVHD (33.3% vs 13.6%; *P* = .097) and the median time to neutrophil (20 days vs 17 days; *P* = .054) and platelet engraftment (34 days vs 27.5 days; *P* = .133) were not significantly different. All patients achieved neutrophil engraftment; however, 1 patient later developed secondary graft failure requiring CD34+ stem cell boost. Platelet engraftment occurred in all patients within 35–86 days after alloHCT except 5 who died before engraftment.

**Table 2.** Characteristics of HHV-6B Reactivation and Outcomes of Treated vs Observed Patients

Outcomes	Observation (n = 37)	Treated With Antivirals (n = 21)	*P* Value
High-level DNAemia ≥10 000 copies/mL (peak), No. (%)	15 (40.5)	18 (85.7)	<.001
Median onset of DNAemia (IQR), d from HCT	26 (24.0–32.5)	23 (18.5–26.5)	.022
Median peak viral load (IQR), copies/mL	6840 (1670–35 250)	44 900 (20 200–210 000)	<.001
Median peak viral load (IQR), log_10_	3.84 (3.22–4.54)	4.65 (4.29–5.32)	<.001
Median time to peak viral load (IQR), d	27 (24.0–32.5)	25 (20.0–32.0)	.303
LP performed, No. (%)	10 (27)	16 (76.2)	<.001
HHV-6 in CSF, No. (% of LP)	2 (20)	4 (25)	1.00
CNS symptoms, No. (%)	4 (10.8)	7 (33.3)	.077
Median onset of CNS symptoms (IQR), d from HCT	20 (13.75–44.25)	26 (20.00–33.00)	.788
Median time to clearance of HHV-6 DNA (IQR), d	12 (7.5–21.0)	14 (7.0–15.0)	.782
Second reactivation episode, No. (%)	0 (0)	4 (19)	.014
Grade II–IV acute GVHD, No. (%)	13 (35.1)	12 (57.1)	.104
Grade III/IV acute GVHD, No. (%)	5 (13.6)	7 (33.3)	.097
Median time to neutrophil engraftment (IQR), d from HCT	17 (15.0–20.5)	20 (16.0–30.5)	.054
Median time to platelet engraftment (IQR), d from HCT	27.5 (22.5–35.0)	34.0 (26.0–48.0)	.133
Day 100 NRM, No. (%)	4 (10.8)	8 (38.1)	.020

Abbreviations: CNS, central nervous system; GVHD, graft-vs-host disease; HCT, hematopoietic cell transplant; HHV-6B, human herpesvirus 6B; IQR, interquartile range; LP, lumbar puncture; NRM, nonrelapse mortality.

A univariate analysis for day 100 NRM is presented in Table 3. CNS symptoms (HR, 6.15; 95% CI, 1.98–19.11; *P* = .002), receipt of treatment (HR, 3.90; 95% CI, 1.17–12.98; *P* = .026), female sex (HR, 3.56; 95% CI, 1.07–11.84; *P* = .038), and the presence of grade III/IV acute GVHD (HR, 3.69; 95% CI, 1.17–11.70; *P* = .026) were factors associated with higher day 100 NRM after alloHCT. Additionally, for each point increase in SOFA score, day 100 NRM increased (HR, 1.50; 95% CI, 1.12–1.99; *P* = .006). Achievement of platelet engraftment was associated with lower day 100 NRM (HR, 0.27; 95% CI, 0.08–0.88; *P* = .030). In the multivariate analysis (Table 4), only the presence of CNS symptoms was an independent predictor of day 100 NRM (HR, 4.11; 95% CI, 1.27–13.34; *P* = .018).

**Table 3.** Univariate Cox Proportional Hazards Model for Day 100 Nonrelapse Mortality

Variables	Status	*P* Value	HR		
			Point	Lower	Upper
Treated	No	Reference			
	Yes	.026	3.90	1.17	12.98
Age (per 10-y increase)		.061	1.67	0.98	2.85
Sex	Male	Reference			
	Female	.038	3.56	1.07	11.84
Race	Not White	Reference			
	White	.094	5.74	0.74	44.52
SOFA score (per 1-point increase)		.006	1.50	1.12	1.99
Peak viral load	<10^4^ copies/mL	Reference			
	≥10^4^ copies/mL	.87	1.10	0.35	3.47
Underlying malignancy	Nonmyeloid malignancy	Reference			
	Myeloid malignancy	.38	1.99	0.44	9.07
GVHD prophylaxis	PTCy/tacrolimus/MMF	Reference			
	PTCy/sirolimus/MMF	.66	1.34	0.36	4.97
CMV status	D+/R+	Reference			
	D+/R–	.62	0.59	0.07	4.78
	D−/R+	.42	0.53	0.11	2.53
	D−/R–	.77	0.79	0.16	3.81
Reason for HHV-6 evaluation	Serial	Reference			
	Symptomatic	.12	2.82	0.76	10.43
Platelet engraftment	No	Reference			
	Yes	.030	0.27	0.08	0.88
Maximum acute GVHD grade	Grade 0–II	Reference			
	Grade III/IV	.026	3.69	1.17	11.70
CNS symptoms	No	Reference			
	Yes	.002	6.15	1.98	19.11

Abbreviations: CMV, cytomegalovirus; CNS, central nervous system; D, donor; GVHD, graft-vs-host disease; HHV-6, human herpesvirus 6; HR, hazard ratio; MMF, mycophenolate mofetil; PTCy, post-transplant cyclophosphamide; R, recipient; SOFA, Sequential Organ Failure Assessment.

**Table 4.** Multivariate Cox Proportional Hazards Model for Day 100 Nonrelapse Mortality

Variables	Levels	*P* Value	HR		
			Point	Lower	Upper
Sex	Male	Reference			
	Female	.082	2.98	0.87	10.20
Platelet engraftment	No	Reference			
	Yes	.078	0.32	0.09	1.13
CNS symptoms	No	Reference			
	Yes	.018	4.11	1.27	13.34

Abbreviations: CNS, central nervous system; HR, hazard ratio.

### HHV6 Detection in the CSF

Twenty-six patients (44.8%) had a lumbar puncture performed doing the study period. LP occurred more frequently in treated patients (76.2% vs 27%; *P* < .001), but detection of HHV-6B in CSF was similar between groups. Six (10.3%) patients had HHV-6B DNA detected in CSF, of whom 4 received treatment; these are described in Supplementary Table 2. Only 1 patient had CNS symptoms and detectable HHV-6B on CSF evaluation. This patient had adrenoleukodystrophy with baseline dysarthria and mobility issues before transplant and developed acute mutism progressing to bilateral upper extremity weakness. The initial lumbar puncture did not detect HHV-6B DNA. A repeat lumbar puncture 16 days later also remained negative for HHV-6B DNA in CSF, but a subsequent LP obtained 27 days after onset of altered mental status detected HHV-6B.

Overall, 11 patients (19%) had documented CNS symptoms, and of those 8 (72.7%) had CSF evaluation. None had detectable HHV-6B DNA in the CSF at the time of initial lumbar puncture, and no cases of definitive HHV-6B encephalitis were identified. Of patients with CNS symptoms, 8 (72.7%) underwent magnetic resonance imaging of the brain. None had acute abnormal findings to potentially attribute to HHV-6B except the patient with adrenoleukodystrophy.

### Impact of High Level HHV-6B DNAemia on Outcomes

Patients with high-level DNAemia (≥10^4^ copies/mL in plasma) were more likely to receive treatment (Supplementary Table 3). The high-level and lower-level DNAemia groups had similar proportions of patients with presence of CNS symptoms (21.2% vs 16.0%; *P* = .742) and grade III/IV acute GVHD (24.2% vs 16.0%; *P* = .443). In addition, these 2 groups did not differ in day 100 NRM (21.2% vs 20.0%; *P* = .910). No association to time to neutrophil engraftment (18 days vs 18 days; *P* = .987) or platelet engraftment (27 days vs 30.5 days; *P* = .316) was observed.

## DISCUSSION

In this single-center retrospective study of patients with HHV-6B reactivation following haploidentical alloHCT, we observed no significant differences in transplant-related outcomes between treated vs observed patients. The presence of CNS symptoms in patients with HHV-6B reactivation was the only factor associated with day 100 NRM after alloHCT in multivariable analysis. The overall incidence of HHV-6B encephalitis following alloHCT may be as high as 2.3%, but for cord blood transplant it has been reported to be as high as 9.9% [3, 13, 26]. Detection of HHV-6B DNA in CSF alone is insufficient for diagnosis and can occur in patients without CNS dysfunction [27]. Another study of 208 alloHCT recipients prospectively monitored weekly for HHV-6 DNA in plasma found 4 cases of proven encephalitis and 5 cases of possible or probable encephalitis due to HHV-6 [28]. In our study, 26 patients underwent lumbar puncture and CSF analysis, with HHV-6B DNA detected in 6 (23.1%) of those tested. Only 1 patient had CNS dysfunction at the time of detection; however, the role of HHV-6B was unclear due to underlying adrenoleukodystrophy. Three patients with CNS dysfunction did not have a lumbar puncture, so it would be difficult to exclude HHV-6B as the causative etiology. Based on our data, the incidence of HHV-6B encephalitis may be lower than that reported for cord blood transplant and potentially in line with historical populations; however, a larger independent cohort study with prospective monitoring could better evaluate this risk. Furthermore, CNS symptoms in the alloHCT setting have wide differential diagnosis including presence of other infections or transplant-associated thrombotic microangiopathy, which are well-established risk factors for NRM.

A large study of 404 alloHCT patients monitored for viral kinetics of DNA viruses in plasma obtained weekly for CMV monitoring identified 224 episodes of HHV-6B [29]. A blip, defined as detection ≤1 week, occurred in nearly half of patients, and no episodes of end-organ disease were found in that population, whereas sustained DNAemia, defined as >4 weeks of detectable DNA, was found in less than a quarter of patients [29]. Of those, HHV-6 disease only developed in 3.3% of 90 episodes of sustained DNAemia [29]. Isolated DNAemia poorly predicts onset of end-organ disease. In our population, 13 (22.4%) patients developed DNAemia lasting ≤1 week, and only 7 (12.1%) patients had DNAemia for >4 weeks, with 4 additional patients not having at least 1 follow-up level evaluated >4 weeks after onset of DNAemia. A second episode of HHV-6B DNAemia was more common in treated patients (19% vs 0%; *P* = .014), though its significance is unclear.

We observed no clinical outcome differences between patients with high-level DNAemia. High-level DNAemia of ≥10^4^ copies/mL in plasma had previously been considered sensitive to rule out HHV-6 encephalitis (Supplementary Table 3) [4]. In that study, less than half of patients without high-level DNAemia and CNS dysfunction had CSF assessment, and subsequent studies have found HHV-6B encephalitis and PALE absent high-level reactivation [12, 14, 30]. Viral loads rise rapidly within 48 hours of onset of encephalitis, limiting the utility of PCR monitoring if it cannot be performed in real time [1, 4]. While patients with high-level DNAemia more frequently received treatment in our study, there was no significant difference in multiple evaluated transplant-related outcomes. Similar findings have been reported in high-risk populations of umbilical cord blood transplant recipients [8]. Despite the institutional protocol, only 23 of 33 (69.7%) patients with high-level DNAemia underwent LP, often due to cytopenias or procedural risk assessment.

While we observed an increased day 100 NRM in treated patients, this was not significant in a multivariate Cox proportional hazards model. Baseline Karnofsky Performance Scale was not significantly different between groups; therefore, the SOFA score was utilized as treatment may have been initiated due to higher clinical acuity of the patient. However, no significant difference in SOFA scores was found between treated and observed patients. When evaluating day 100 NRM, each point of increase in the SOFA score was associated with higher mortality in the univariate analysis, but this lost significance in the multivariate model. There may be additional confounding variables that we were unable to detect within the limitations of this retrospective study.

When considering the subgroup of 31 symptomatic patients, the day 100 NRM was 60% in treated patients as compared with 14.3% in those clinically observed (*P* = .015). However, there are substantial potential confounders and limitations of this study, and, as noted, in vivo attribution of symptoms to HHV-6B is difficult to prove. In the study by Noviello et al., 52 of 130 (40%) patients with HHV-6 reactivation developed end organ disease as defined by the ECIL guidelines [28]. However, as noted in the guideline, many of those potential disease states are only based on weak or moderate in vitro or in vivo evidence, so it would be difficult to prove causation [9]. In our study, we did not identify any definitive cases of HHV-6B and end organ disease. A key limitation of our study is that haploidentical transplant recipients were not uniformly prospectively monitored. Furthermore, despite an institutional protocol for HHV-6B monitoring and treatment, the decision to serially monitor patients and/or initiate therapy was ultimately determined by the treating physician, which introduces potential for bias.

The implications and impact of HHV-6B following haploidentical alloHCT remain poorly understood, and guidelines note that further evidence is needed. There are limited data regarding preemptive therapy for HHV-6B DNAemia in haploidentical alloHCT recipients. One study evaluated the use of preemptive foscarnet of 60–90 mg/kg/d for 11 patients with HHV-6 DNAemia at a threshold of ≥1000 copies/mL or >188 copies/mL in the presence of cytopenia following haploidentical alloHCT and reported no side effects attributable to therapy [31]. Its lack of a comparative group limits the ability to draw conclusions. In our study, we observed a high rate of complications from antiviral therapy, with >40% of patients developing an AKI by KDIGO definitions after initiation of foscarnet therapy, and of those, 31.6% of AKI cases were attributed to drug therapy by the treating physician. Dosages utilized for treatment of and prophylaxis for HHV-6B differ across studies and may have different risks of nephrotoxicity.

Acute GVHD has a unique relationship with HHV-6 reactivation and is an established risk factor [2]. In this study, 12 (20%) patients had grade III/IV acute GVHD, similar to historical cohorts. There were no differences in GVHD among treated vs observed patients. Despite the benefits of PTCy-based approaches, there are reported higher rates of DNA virus reactivations including HHV-6B as well as potential infections that require ongoing investigation [28, 32, 33]. The Center for International Blood and Marrow Transplant Research does not collect data on institutional practices for viral detection; therefore, it is not possible to confirm whether the HHV-6B testing in this population was driven by clinical concerns or more aggressive screening. Acknowledging the limitations of the retrospective nature of the study, we did not observe that treatment for HHV-6B DNAemia impacted transplant-related outcomes. Further controlled studies are required to confirm this study's findings and characterize the impact of HHV-6B reactivation and treatment in haploidentical transplantation.

## Supplementary Material

ofae564_Supplementary_Data

## Supplementary Data

Supplementary materials are available at *Open Forum Infectious Diseases* online. Consisting of data provided by the authors to benefit the reader, the posted materials are not copyedited and are the sole responsibility of the authors, so questions or comments should be addressed to the corresponding author.
