# Supplementary material for: Outcomes of Haploidentical Stem Cell Transplant Recipients With HHV-6B Reactivation
Source: Open Forum Infect Dis. 2024 Sep 26;11(10):ofae564. doi: 10.1093/ofid/ofae564 (PMC11475747; doi:10.1093/ofid/ofae564)
Supplement: ofae564_Supplementary_Data [file ofae564_supplementary_data.docx]

Supplemental Table 1: Comparison of Engraftment Status, acute GVHD and Steroid dosing at onset of viremia

| Variable | **Observation (n=37)** | **Treated with Antivirals (n=21)** | ***P* value** |
| --- | --- | --- | --- |
| ANC < 500/mm^3^ | 2 (5.4) | 9 (42.9) | <0.001 |
| Platelet Engraftment Achieved | 22 (59.5) | 18 (85.7) | 0.038 |
| Acute GVHD Grade 2-4 | 6 (16.2) | 2 (9.5) | 0.698 |
| Acute GVHD Grade 3-4 | 2 (5.4) | 1 (4.8) | 1 |
| Receipt of >0.5 mg/kg prednisone equivalent | 5 (13.5) | 0 (0) | 0.148 |

ANC, absolute neutrophil count; GVHD, graft-versus-host disease

Supplemental Table 2: Characteristics of Patients with Detection of HHV-6B in Cerebrospinal Fluid

| Patient | Age, years | Sex | Malignancy | Conditioning Regimen | GVHD Prophylaxis | Engraftment, Days after HCT | Grade 3/4 Acute GVHD | HHV-6B Reactivation, Days after HCT | HHV-6B plasma peak, copies/mL | HHV-6B CSF peak, copies/mL | CNS Symptoms | Imaging | Treatment | Day 100 NRM |
| --- | --- | --- | --- | --- | --- | --- | --- | --- | --- | --- | --- | --- | --- | --- |
|  |  |  |  |  |  |  |  |  |  |  |  |  |  |  |
| 1 | 63 | Male | MPS | Fludarabine, Busulfan | PTCy/Sirolimus/MMF | 30 | No | 24 | 135000 | 2370 | No | No findings on non-contrasted CT head | Yes, Foscarnet | No |
| 2 | 38 | Male | Sickle Cell Disease | Fludarabine, Cyclophosphamide, TBI, ATG | PTCy/Sirolimus/MMF | 14 | No | 17 | 135000 | N/A^a^ | No | No acute findings on MRI brain | No | No |
| 3 | 75 | Male | MDS | Fludarabine, Cyclophosphamide, TBI | PTCy/Sirolimus/MMF | 20 | No | 27 | 316000 | 50000 | No | N/A | Yes, Foscarnet | Yes |
| 4 | 55 | Female | AML | Fludarabine, Busulfan | PTCy/Sirolimus/MMF | 14 | No | 23 | 718000 | 8540 | No | N/A | No | No |
| 5 | 20 | Male | Adrenoleukodystrophy | Fludarabine, Busulfan | PTCy/Sirolimus/MMF | 16 | No | 18 | 36800 | 1160^b^ | Yes, acute mutism and upper extremity weakness | Worsening T2 hyperintensities in cerebellum, midbrain, thalamus and internal capsule on MRI brain compared to pre-transplant MRI obtained 4 months prior | Yes, Foscarnet | No |
| 6 | 67 | Male | AML | Fludarabine, Cyclophosphamide, TBI | PTCy/Tacrolimus/MMF | 18 | No | 19 | 15600 | 3000 | No | N/A | Yes, Foscarnet | No |

GVHD, graft-versus-host disease; HCT, hematopoietic cell transplant; CSF, cerebrospinal fluid; CNS, central nervous system; NRM, non-relapse mortality; MPS, myeloproliferative syndrome; PTCy, post-transplant cyclophosphamide; MMF, mycophenolate mofetil; TBI, total body irradiation; ATG, antithymocyte globulin; MDS, myelodysplastic syndrome, AML, acute myeloid leukemia

^a^detected, but not quantifiable

^b^ Not detected on initial two lumbar punctures

Supplemental Table 3: Comparison of Clinical Features and Outcomes of Patients with High Level DNAemia

| **Outcome** | **Peak Viral Load ≥ 10^4^ copies/mL n=33** | **Peak Viral Load < 10^4^ copies/mL n=25** | ***P* Value** |
| --- | --- | --- | --- |
| Antiviral Treatment Administered, n (%) | 18 (54.5) | 3 (12.0) | **<0.001** |
| Grade III-IV acute GVHD, n (%) | 8 (24.2) | 4 (16.0) | 0.443 |
| CNS Symptoms, n (%) | 7 (21.2) | 4 (16.0) | 0.742 |
| Day 100 Non-Relapse Mortality, n (%) | 7 (21.2) | 5 (20.0) | 0.910 |
| Time to Neutrophil Engraftment (days from HCT) (IQR) | 18 (15.00-23.50) | 18 (15.50-22.00) | 0.987 |
| Time to Platelet Engraftment (days from HCT) (IQR) | 27.0 (23.00-40.00) | 30.50 (25.00-49.00) | 0.316 |

GVHD, graft-versus-host disease; CNS, central nervous system; HCT, hematopoietic cell transplant
